# Evaluation of hypoglycemic effect, safety and immunomodulation of *Prevotella copri* in mice

**DOI:** 10.1038/s41598-021-96161-6

**Published:** 2021-10-28

**Authors:** Phebe Verbrugghe, Jón Brynjólfsson, Xingjun Jing, Inger Björck, Frida Hållenius, Anne Nilsson

**Affiliations:** 1 Food Technology, Engineering and Nutrition, Lund University, PO Box 124, 221 00 Lund, Sweden; 2 Food for Health Science Centre, Lund University, PO Box 124, 221 00 Lund, Sweden; 3 Present Address: ProPrev AB, 254 40 Helsingborg, Sweden

**Keywords:** Health care, Metabolic disorders, Bacteria, Bacterial host response, Applied microbiology

## Abstract

The gut bacterium *Prevotella copri* (*P. copri)* has been shown to lower blood glucose levels in mice as well as in healthy humans, and is a promising candidate for a next generation probiotic aiming at prevention or treatment of obesity and type 2 diabetes. In this study the hypoglycemic effect of live *P. copri* was confirmed in mice and pasteurization of *P. copri* was shown to further enhance its capacity to improve glucose tolerance. The safety of live and pasteurized *P. copri* was evaluated by a 29-day oral toxicity study in mice. *P. copri* did not induce any adverse effects on body growth. General examination of the mice, gross pathological and histological analysis showed no abnormalities of the vital organs. Though relative liver weights were lower in the pasteurized (4.574 g ± 0.096) and live (4.347 g ± 0.197) *P. copri* fed groups than in the control mice (5.005 g ± 0.103) (p = 0.0441 and p = 0.0147 respectively), no liver biochemical marker aberrations were detected. Creatinine serum levels were significantly lower in mice fed with live (p = 0.001) but not pasteurized (p = 0.163) *P. copri* compared to those of control mice. Haematological parameter analysis and low plasma Lipopolysaccharide Binding Protein (LBP) levels ruled out systemic infection and inflammation. Immunomodulation capacity by *P. copri* as determined by blood plasma cytokine analysis was limited and gut colonisation occurred in only one of the 10 mice tested. Taken together, no major adverse effects were detected in *P. copri* treated groups compared to controls.

## Introduction

The prevalence of prediabetes, a condition characterised by blood glucose levels between normal and diabetic levels, is estimated between 15.8% and 36.0% (depending on the diagnostic criteria used) worldwide and is rapidly increasing. Of the individuals with prediabetes as many as 70% will eventually develop diabetes^1^. Metabolic diseases such as type 2 diabetes and obesity are major causes of death and disability worldwide. According to the International Diabetes Federation nearly 9.3% of the global population is affected by diabetes and the number is rising^2^. This causes a huge financial burden on the health care system globally and indicates the urgent need for interventions to prevent, delay and treat these diseases and their progression to debilitating complications (i.e. stroke, acute myocardial infarction, amputation or death from hyperglycaemic crisis).

Probiotics—originally introduced to improve intestinal health, are increasingly being considered also as preventative agents or therapeutics for type 2 diabetes and obesity^3^. Emerging evidence points to a central role for gut microbiota in host metabolism, for example blood glucose homeostasis and appetite regulation^4,5^. Profound differences in gut microbiota composition have been observed between obese and lean subjects and between healthy and type 2 diabetes (T2D) subjects, and animal experiments have shown a causal relationship between gut microbiota and disease^6^. In human metabolic syndrome subjects, furthermore, faecal transplantation with lean donor faeces has been shown to improve insulin sensitivity which was associated with changes in intestinal microbiota^7^.

A recent study in healthy humans showed a beneficial effect of barley kernel-based bread on glucose metabolism and this was linked to the composition of the gut microbiota. 16S rRNA gene sequencing and metagenomic analysis revealed that the gut microbiota in the subjects that responded to the barley kernel diet with improved glucose tolerance was enriched in *P. copri.* Germ free and normal wild type mice gavaged with *P. copri* showed an improved glucose tolerance pointing to a causal role^8^. This can possibly be attributed to *P. copri*’s ability to enhance hepatic glycogen storage^8^ and to produce succinate, a tricarboxylic acid (TCA) cycle intermediate, by bacterial fermentation of the barley dietary fibers. Succinate is responsible for glucose homeostasis through modulating intestinal gluconeogenesis^9^. Enrichment of *Prevotella copri* after intake of barley kernels was also linked to an increase in the gut hormone GLP-1 and lower hunger sensations^10^. In line with these findings *P. copri* has been identified as a potential candidate for a next generation probiotic to prevent and treat metabolic diseases^11^.

*P. copri*, a commensal obligate anaerobic gram-negative bacterium (for general characteristics see Table 1), is one of the most prevalent bacteria in the human gastrointestinal tract^12^. In 10–25% of healthy Americans and European individuals, the relative abundance of *Prevotella* in the stool is > 10%^13^ and of the 5 *Prevotella* species reported in the human intestine *P. copri* is the most abundant^14^. *Prevotella* is a biomarker of diet, lifestyle and health status^15^ and *P. copri* abundance has been linked to vegetable and fiber-rich low protein diets^16–18^ which are more commonly consumed by non-Western subjects^18,19^. Decreased levels of *P. copri* were observed in obese African women^20^ and *Prevotella* abundance can be used as a predictor of weight loss success in healthy, overweight adults consuming a whole-grain diet^21^. While increased levels of *P. copri* were reported in patients with newly onset untreated rheumatoid arthritis^22^ and irritable bowel syndrome^23^, decreased levels of *P. copri* were detected in patients with neurodegenerative diseases^24^ and childhood dermatitis^25^.

**Table 1:** General characteristics of *P. copri.*

Phylum	Class	Order	Family	Genus	Species
Bacteroidetes	Bacteroidia	Bacteroidales	Prevotellaceae	*Prevotella*	*Prevotella copri*
Type strain	DSM 18205 (CB7)				
Biosafety level	1				
Origin	Faecal sample from a healthy adult volunteer				
Characteristics	Gram negative, rod-shaped, anaerobic and non-spore forming bacteria				
Growth conditions	Medium	Schaedler Agar with horse blood			
	Temperature	37 °C			
	Atmosphere	5% H_2_, 10% CO_2_, 85% N_2_			

Taken together, *P. copri* shows potential as next generation probiotic to prevent or treat metabolic related diseases, and appears to affect also other pathologies. To justify human consumption, however, its safety needs to be validated, especially considering its ambiguous association with health status^26^. In this study, therefore, the effects of *P. copri* administration were evaluated in mice. Mice were gavaged daily with *P. copri* for 29 consecutive days. Potential pathological effects were studied by analysis of weight, and evaluation of haematological, histological and biochemical parameters. Immunomodulation, translocation and colonisation by the bacteria were also determined.

## Results

### Oral glucose tolerance test in C57BL/6J mice gavaged with *P. copri*

**Figure 1:**
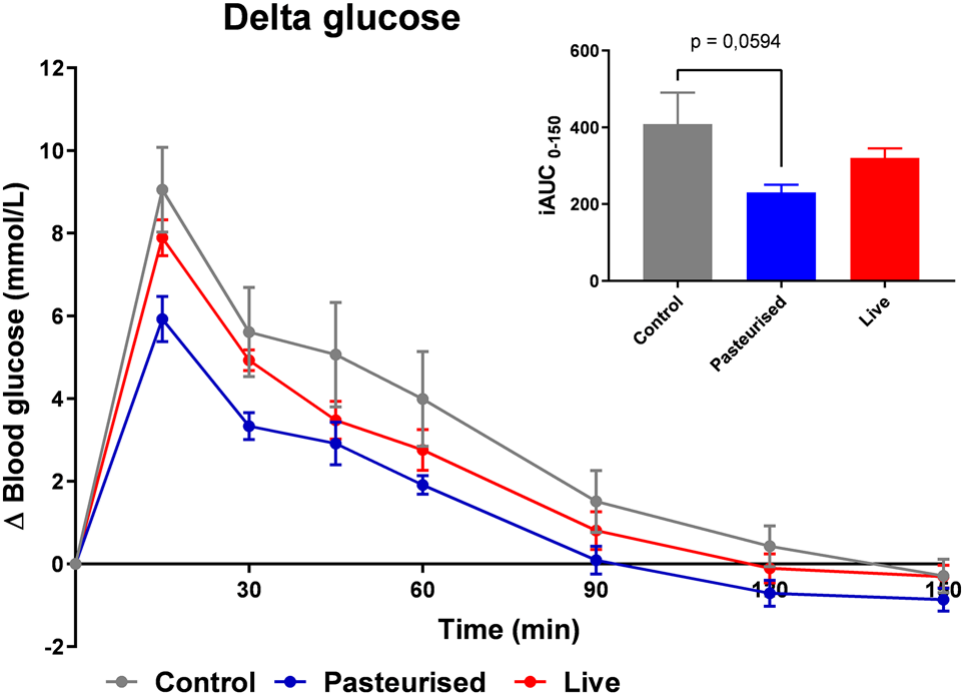
Beneficial Effects of *P.* *copri* on Glucose Metabolism in Mice. C57BL/6J mice were gavaged with 5 × 10^6^ live *P. copri* daily for 7 consecutive days. An oral glucose tolerance test (OGTT) was performed on day 7 showing that both live and pasteurized *P. copri* improve glucose tolerance (n = 10).

Both the group of mice treated with pasteurized and the group treated with live *P. copri* showed improved glucose tolerance compared to the control (PBS/glycerol only) group, with a more pronounced effect following gavage with the pasteurized bacteria than the live bacteria (Fig. 1a) (area under the curve (p = 0.0594 for control versus pasteurized *P. copri*; Fig. 1b).

### General health status and body weight changes after *P. copri* administration

**Figure 2:**
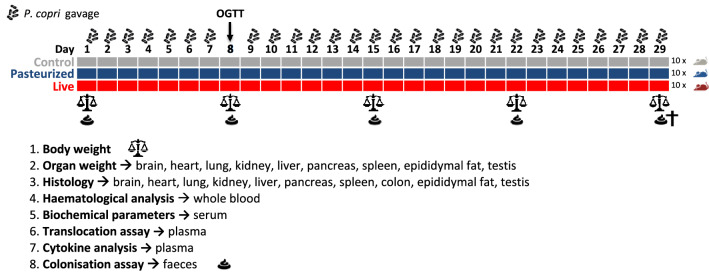
Experimental setup of the mouse study. This figure has been created using Excel office 365 (office.com). The icons were obtained from the following sources: ‘https://pngimage.net/poop-icon-png-3/’ by perfectly posh and ‘https://www.iconsdb.com/black-icons/scales-icon.html’ by Icons8.com, Creative Commons Attribution-NoDerivs 3.0.

**Figure 3:**
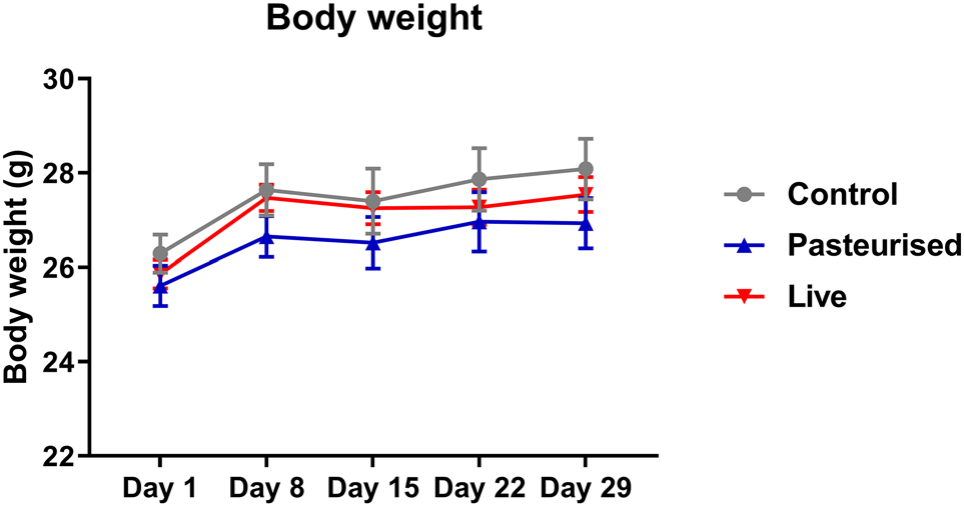
Body weights before the start of gavaging (day 1) and at day 8, 15, 22 and 29 (n = 10).

No death or treatment-related signs of toxicity were observed in any of the animals during the 29 days. General appearance and behavior of the animals were similar for all groups throughout the study. The skin appeared normal (no ruffled fur or skin lesions) and no diarrhea was observed. All mice were active and curious (open-field activity), exhibited normal grooming activity and no abnormal locomotion, hyperactivity or lethargy (sluggish behavior, hunched posture) was noted. Bodyweight was measured five times during the experiment (before the gavaging started, after week 1, 2 and 3 and on the day of sacrifice) (Fig. 2). All groups gained weight at similar rates and none of the treatment groups showed abnormal bodyweight gain/loss (Fig. 3).

### Macroscopic and histopathological examination

**Figure 4:**
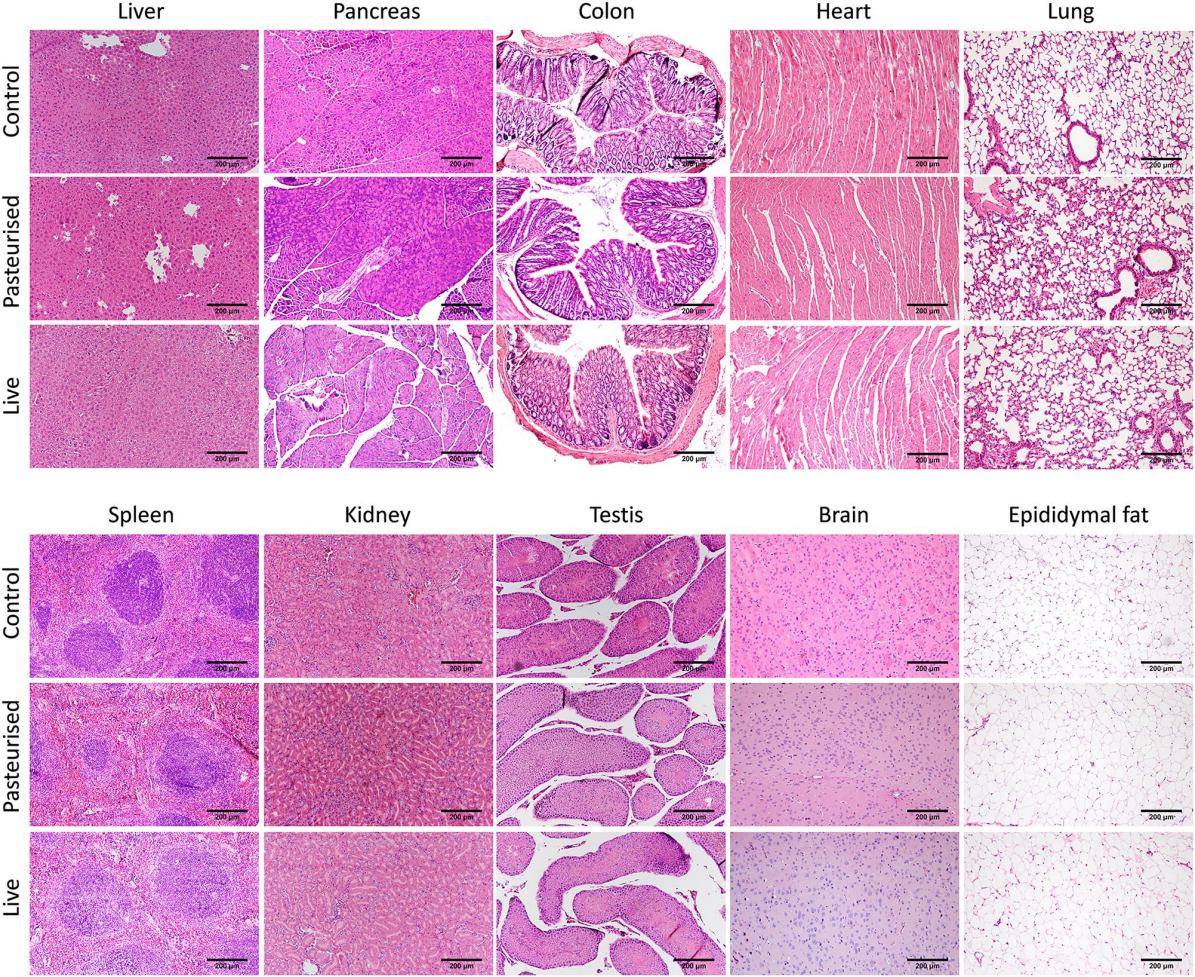
Representative histological haematoxylin–eosin stained sections of murine organs of control mice (top) and after administration of pasteurized (middle) or live (bottom) *P. copri* (liver, pancreas, colon, heart, lung, spleen, kidney, testis, brain and epipidymal fat). Scale bar = 200 μm.

No significant difference in organ weight relative to body weight between control and *P. copri* (pasteurized or live) fed mice was observed for pancreas, heart, lung, brain, spleen, epididymal fat, kidneys or testis weight relative to body weight (Table 2). The liver weight of mice which received pasteurized (4.574 g ± 0.096) and live *P. copri* (4.347 g ± 0.197) was lower than the relative liver weight of the control mice (5.005 g ± 0.103), p = 0.0441 and p = 0.0147 respectively. Gross pathological and histopathological examination revealed no signs of inflammation, degeneration or necrosis of liver, pancreas, colon, heart, lung, spleen, kidney, testis, brain or epididymal fat (Fig. 4).

**Table 2:** Relative organ weights after oral administration of *P. copri* for 29 days.

Organ weight	Control	Pasteurized	Live
Pancreas/BW (%)	0.823 ± 0.057	0.847 ± 0.063	0.955 ± 0.045
Heart/BW (%)	0.536 ± 0.011	0.534 ± 0.018	0.573 ± 0.028
Lung/BW (%)	0.531 ± 0.024	0.633 ± 0.036	0.619 ± 0.033
Spleen/BW (%)	0.246 ± 0.012	0.268 ± 0.013	0.276 ± 0.010
Kidneys/BW (%)	1.206 ± 0.034	1.237 ± 0.029	1.266 ± 0.031
Liver/BW (%)	5.005 ± 0.103	4.574 ± 0.096*	4.347 ± 0.197*
Testes + epididymes/BW (%)	0.928 ± 0.032	0.888 ± 0.039	0.922 ± 0.019
Epididymal fat/BW (%)	1.178 ± 0.078	1.177 ± 0.081	1.150 ± 0.082
Brain/BW (%)	1.222 ± 0.055	1.192 ± 0.025	1.169 ± 0.013

Values are presented as mean ± SEM of 10 replicates.

BW body weight.

*Mean values were significantly different from the control group (p-value < 0.05).

### Haematological analysis and serum biochemistry profile

**Figure 5:**
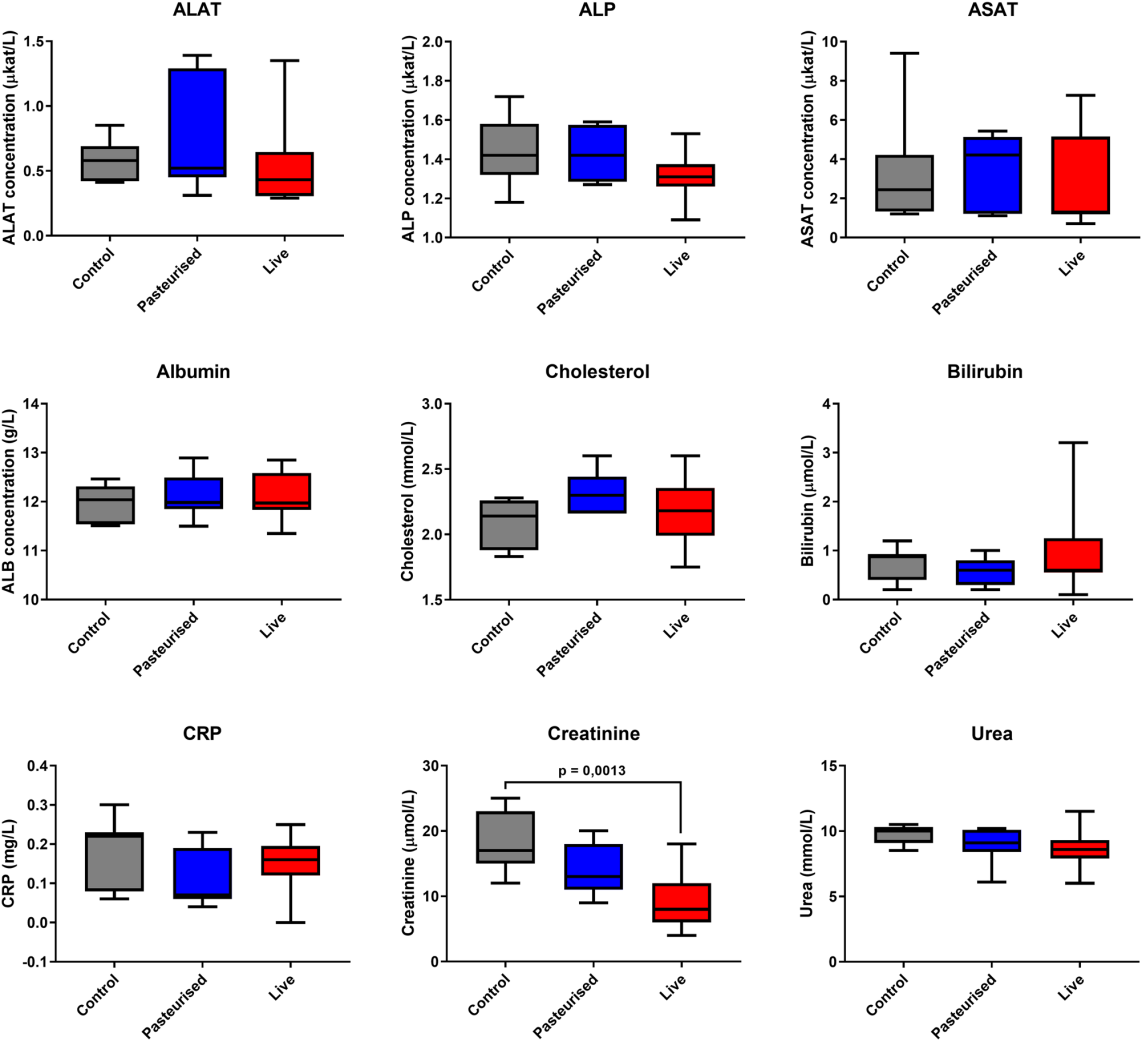
Serum biochemical markers after oral administration of *P. copri* for 29 days. ALAT, alanine aminotransferase; ASAT, aspartate aminotransferase; ALP, alkaline phosphatase; ALB, Albumin; CRP, C-reactive protein. Samples which showed signs of haemolysis were not included in the analysis. Control and pasteurized *P. copri* n = 7, live *P. copri* n = 9.

Haematological parameters show the total count of white blood cells and their sub-types (lymphocytes, monocytes, neutrophils). No significant alterations were observed using one-way ANOVA for total blood count of white blood cells (p = 0.663), neutrophils (p = 0.171), monocytes (p = 0.937) or lymphocytes (p = 0.381) between the control and the *P. copri*-fed mice. Platelet counts were also not significantly different between the treatments (p = 0.638, one-way ANOVA) (Table 3). Repeated-dose oral administration of *P. copri* did not result in any significant changes in the serum biochemistry parameters aspartate amino transferase (AST), alanine amino transferase (ALT), alkaline phosphatase (ALP), albumin (ALB), cholesterol, bilirubin, C-reactive protein (CRP) and blood urea nitrogen (BUN), compared with the control group. Creatinine serum levels however, were significantly lower in mice fed with live (p = 0.001) but not pasteurized (p = 0.163) *P. copri* compared to those of control mice (Fig. 5).

**Table 3:** Haematological parameters after oral administration of *P. copri* for 29 days.

Blood cell parameter	Control	Pasteurized	Live
WBC (10^9^/L)	4.098 ± 0.596	4.497 ± 0.494	4.880 ± 0.666
LYM (10^9^/L)	3.301 ± 0,543	3.762 ± 0.410	4.373 ± 0.603
MON (10^9^/L)	0.313 ± 0.080	0.330 ± 0.067	0.294 ± 0.063
NEU (10^9^/L)	0.483 ± 0.127	0.403 ± 0.091	0.214 ± 0.073
MCH (pg)	14.280 ± 0.315	14.250 ± 0.119	14.480 ± 0.111
MCHC (g/dL)	32.500 ± 0.764	33.510 ± 0.254	33.410 ± 0.206
RDWs (fl)	33.290 ± 0.522	32.040 ± 0.302	32.590 ± 0.502
RDWc % (%)	17.420 ± 0.214	17.370 ± 0.157	17.360 ± 0.200
PLT (10^9^/L)	429.100 ± 26.429	447.900 ± 57.510	486.800 ± 40.908
PCT % (%)	0.233 ± 0.016	0.245 ± 0.033	0.263 ± 0.022
MPV (fl)	5.450 ± 0.083	5.460 ± 0.070	5.390 ± 0.069
PDWs (fl)	5.650 ± 0.099	5.830 ± 0.176	5.820 ± 0.184
PDWc % (%)	27.800 ± 0.257	28.230 ± 0.404	28.180 ± 0.419

Values are presented as mean ± SEM (n = 10).

No mean values were significantly different from the control group (p < 0.05).

*WBC* white blood cell count, *LYM* lymphocytes, *MON* monocytes, *NEU* neutrophils, *MCH* mean corpuscular haemoglobin, *MCHC* mean corpuscular haemoglobin concentration, *RDWs* red cell distribution width—SD, *RDWc* red cell distribution width—CV, *PLT* platelet count, *PCT* thrombocrit, *MPV* mean platelet volume, *PDWs* platelet distribution width—SV, *PDWc* platelet distribution width—CV.

### Lipopolysaccharide-binding protein (LBP)

**Figure 6:**
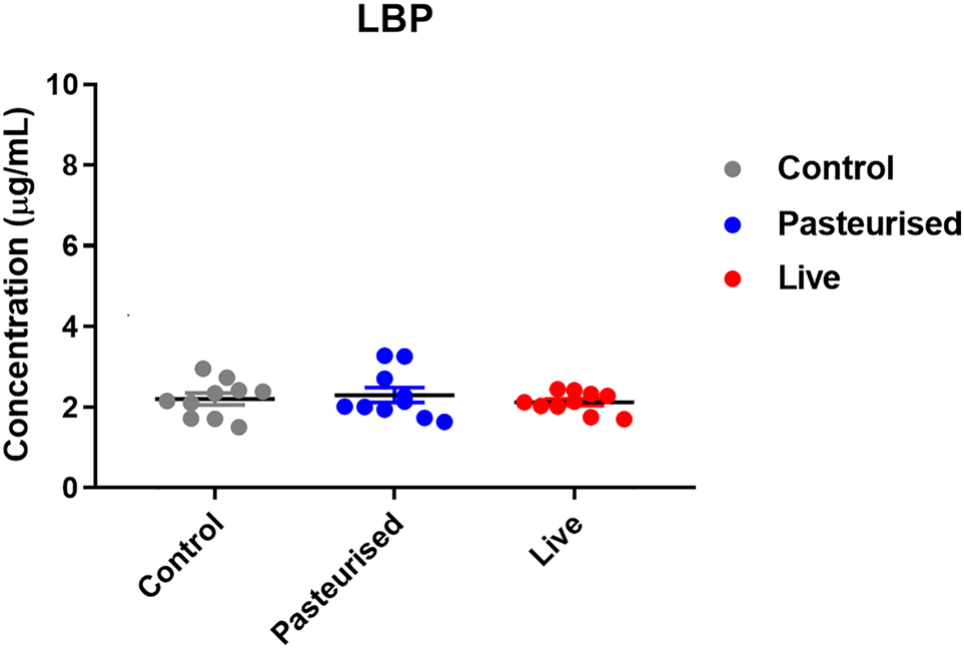
LBP levels in plasma after 29 days of *P. copri* gavage (n = 10).

Plasma LBP levels were not significantly different between the controls and *P. copri* fed groups (control vs pasteurized p = 0.889; control vs live p = 0.920) (Fig. 6).

### Cytokine analysis

**Figure 7:**
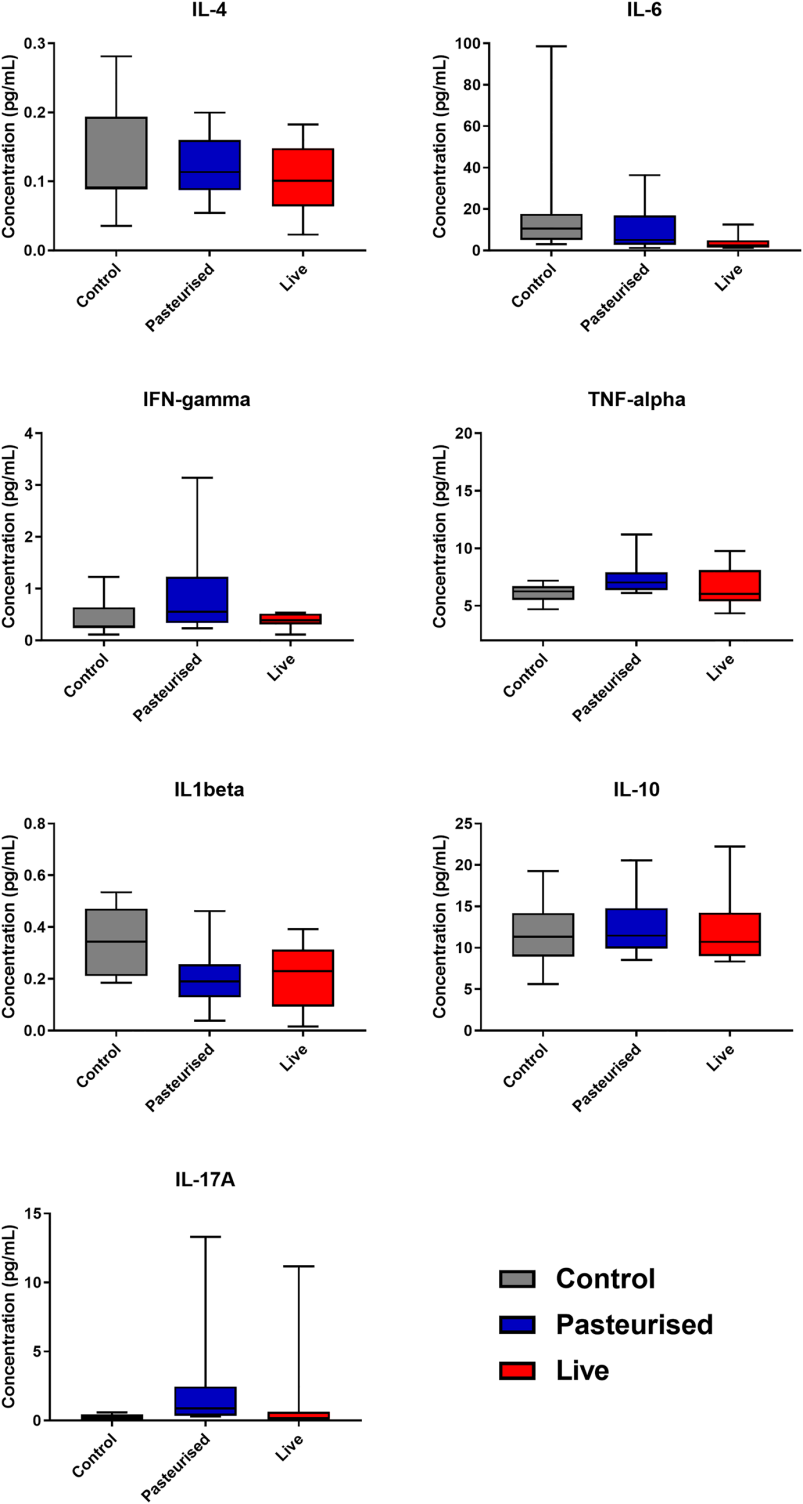
Cytokine levels in plasma after 29 days of *P. copri* gavage (n = 10).

No significant differences between controls and *P. copri* fed mice were detected in blood plasma for IL-4, IL-6, IFN-γ, TNF-α, IL1β, IL-10 or IL-17A (p > 0.05 for all) (Fig. 7).

### Genomic analysis of *P. copri* in stool samples

**Figure 8:**
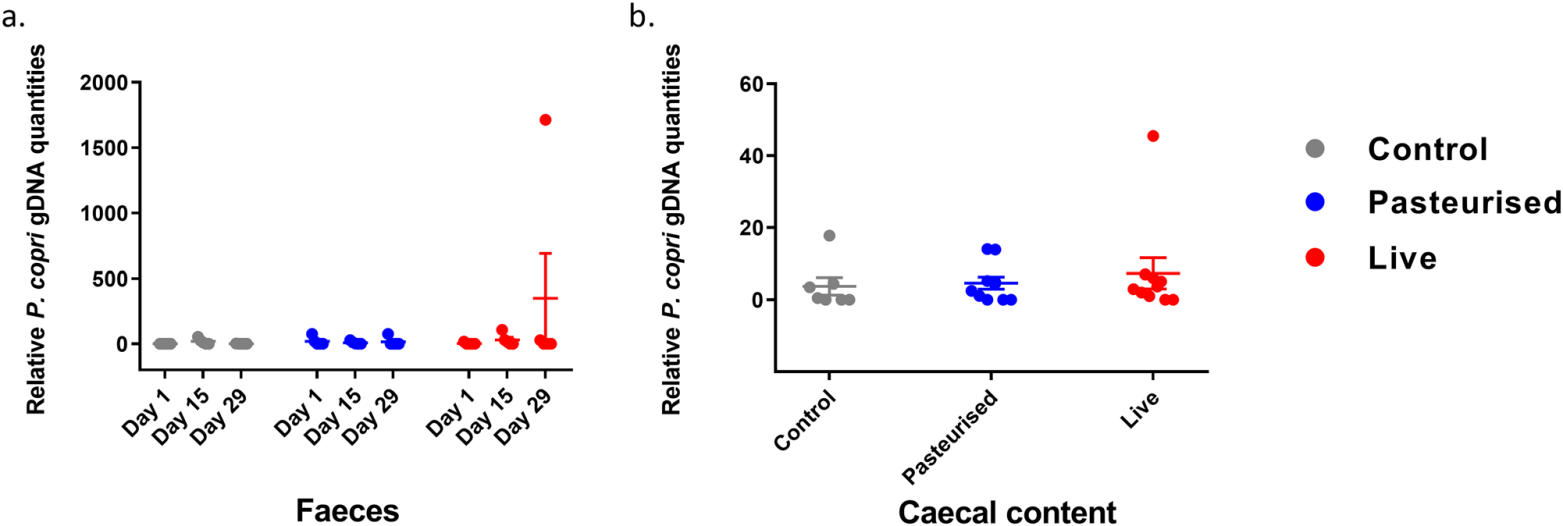
Colonisation assays. Relative genomic DNA content of *P. copri* in caecal content (left) and faeces (right) samples for three treatment groups. Pooled per cage of 2 mice for faeces (n = 5), for faecal content: n = 10.

Faeces were collected and pooled at day 1, day 15 and day 29. Levels of *P. copri* were determined by 16S rRNA gene specific quantitative PCR. *P. copri* gDNA analysis showed a relative increase of the *P. copri* gDNA amounts in the faeces pooled from one cage containing two live *P. copri* fed mice compared to the faeces of the control mice. This elevation in *P. copri* gDNA in the faeces corresponded to the detection of *P. copri* also in the caecal content in one of these 2 mice, indicating the presence of *P. copri* in the intestines of one out of 10 mice (Fig. 8).

## Discussion

The gut bacterium *P. copri* improves glycemic control and has thus been suggested as next generation probiotic^8,11^. For human use, however, its safety needs to be evaluated. In this study the oral toxicity of live and inactivated (pasteurized) *P. copri* was assessed in mice according to the OECD guidelines for the ‘repeated dose 28-day oral toxicity studies’ in rodents^27^, with the setup presented in Fig. 2. Mice were chosen as an experimental model as they are commonly used in human gut microbiota research and their physiology closely resembles that of humans^28^. Pasteurized bacteria as well as live bacteria were tested in the subsequent safety studies as our results showed that pasteurisation enhances *P. copri*’s capacity to improve glucose tolerance (Fig. 1). Inactivation of other probiotics by pasteurization for 30 min at 70 degrees Celsius (a less extreme treatment that in contrast to autoclaving limits denaturation of cellular components) has been shown to partly or fully retain beneficial effects on parameters such as glucose metabolism^29–31^. One hypothesis is that pasteurisation increases access of bacterial ligands to the host receptors. Advantages of using heat inactivated rather than live bacteria include the circumvention of oxygen sensitivity of this anaerobic strain, increased stability and shelf life and elimination of the risks of microbial translocation and infection of the consumer^32^.

Body weights increased at similar rates for all groups indicating no growth abnormalities (Fig. 3). Weight of pancreas, epididymal fat, heart, lung, brain, spleen, kidney or testis relative to body weight was similar in controls and mice treated with *P. copri*, suggesting no adverse effects to these organs. Liver weight relative to bodyweight of mice treated with pasteurized and live *P. copri* however, was lower than that of the controls. To investigate whether the observed decreased liver mass has any functional implications, serum biochemical parameters relevant to liver function and damage were measured. The levels of the liver enzymes AST, ALT and ALP were similar for all groups indicating no liver damage. These enzymes are normally present in liver cells, and to some extent circulating in the blood stream. Upon inflammation or damage of the liver however, the damaged liver cells release elevated levels of these enzymes into the blood stream. Bilirubin, another liver disease marker in the blood stream, is produced from the breakdown of red blood cells. Bilirubin levels were also similar in all groups indicating that *P. copri* administration has no influence on the ability of the liver to remove bilirubin from the blood stream. No significant differences could be observed between the groups for albumin, cholesterol, C-reactive protein (acute-phase protein synthesised by the liver) and urea (a metabolite of amino acids produced in the liver) ruling out hepatoxicity. Creatinine levels however, were significantly lower in the blood of mice fed with live *P. copri* but not pasteurized *P. copri* compared to the controls (Fig. 5). As a by-product of muscle metabolism that is excreted unchanged by the kidneys, serum creatinine is an important indicator of renal function. Lower creatinine levels in the *P. copri* fed groups indicate well-functioning kidneys.

No gross anatomical abnormalities were observed in liver, pancreas, epididymal fat, heart, lung, brain, spleen, kidney, testis, stomach or intestines. Histological analysis of liver, pancreas, colon, heart, lung, spleen, kidney, testis, brain and epididymal fat furthermore showed no microscopic abnormalities (Fig. 4). The epididymal fat cells in the control and pasteurized and live *P. copri* treated groups are all of similar size and do not suggest any fat loss in epididymal tissue. Histology showed no intestinal pathology such as ulcerations of the mucosa or intestinal inflammation that could cause or contribute to augmented intestinal permeability (Fig. 4). Further assessment of bacterial translocation (i.e. the passage of intestinal bacteria or their associated endotoxins from the gut lumen into the circulation that can cause systemic inflammation and distant organ injury) confirmed these histological findings at a molecular level by LBP analysis^33^. There was no significant difference in the LBP levels between the control and *P. copri* fed groups, ruling out gut leakage of lipopolysaccharide (LPS) and metabolic endotoxemia (Fig. 6). LBP is a soluble acute-phase protein mainly produced by the liver that binds to the lipid A portion of bacterial LPS, present on the outer cell wall of gram negative bacteria. It presents LPS to the cell surface LPS receptor complex (CD14, TLR4, MD-2) on immune cells to elicit an acute-phase immunologic response. LBP levels detected were in the normal range for all groups (in the µg/ml range^34^, around 6 µg/ml according to Hycult, the manufacturers of the kit used in this study) and were not indicative of acute phase responses.

To further rule out systemic infection and inflammation, haematological parameters and cytokines were measured in the blood. No significant alterations in blood cell count were observed for total white blood cell count, neutrophils, monocytes or lymphocytes, nor were any significant differences detected in platelet counts (p = 0.638) between the treatments (Table 3). Immunomodulation by *P. copri* determined by cytokine analysis was found to be very limited. Controls and *P. copri* fed mice showed similar blood plasma levels for IL-4, IL-6, IFN-γ, TNF-α, IL1β, IL-10 or IL-17A. Notably, *P. copri* has co-evolved with the human immune system for millennia, confirmed by its presence in archeological gut contents of the 5300 year old European copper age mummy Ötzi and in Mexican coprolite material dating from AD 673 to 768^35^. It has thus evolved presumably harmless strategies to modulate the immune system activities enabling the co-existence of *P. copri* with the human immune system. Interestingly, previous in vitro studies showed that *P. copri* was superior in inducing the Th17 driving cytokines such as IL-6 of bone-marrow-derived dendritic cells (BMDC) compared to other commensal bacteria. These *P. copri* stimulated BMDCs were able to prime native Th cells to produce up to fivefold increased IL-17 levels^36^. In this study however, no significant alterations of IL-17A or IL-6 levels were observed in the plasma of the *P. copri* fed mice (Fig. 7).

A colonisation assay was performed to investigate colonisation potential of *P. copri* in the intestines of the mice. Faeces were collected and pooled per cage before (day 1), in the middle (day 15) and at the end (day 29) of the experiment and caecal content was collected after sacrificing the mice. Levels of *P. copri* were determined by 16S rRNA gene specific quantitative PCR. *P. copri* gDNA was only detected in the faeces collected from one of the live *P. copri* cages (Fig. 8a). The presence of *P. copri* gDNA was narrowed down to one mouse by qPCR of the individual caecal content (Fig. 8b). The absence of detection of *P. copri* in the group that received pasteurized *P. copri* and in 9 out of 10 mice that received live *P. copri* points to degradation of the DNA and/or low quantity of *P. copri*. Notably, the transit time is only 4–6 h in mice (^37^; see Supplementary Fig. S1 for transit time of *P. copri* in particular) while faeces were harvested 24 h after the last gavage in this mouse study. *P. copri* has been reported to colonise the gut of germ-free mice^8^. Given that germ-free mice were not used in this study, it is likely that all niches for *P. copri* were already occupied by other microbes, which could explain the low to absent levels of *P. copri* detected in the faeces.

Since *P. copri* was only discovered in 2007, it has no history of use in the food industry before 1997, and must thus be treated according to the Novel Food Regulation (European Commission, 1997). Further in vitro studies are required to validate the use of *P. copri* as a next generation probiotic. *P. copri* should be screened for the potential presence of genes associated with antibiotic resistance and the minimum inhibitory concentration (MIC)—the lowest concentration of an antimicrobial (like an antifungal, antibiotic or bacteriostatic) drug that will inhibit the visible growth of a microorganism after overnight incubation—should be experimentally determined.

In conclusion, *P. copri* did not induce any adverse effects on growth, cellular blood components, serum biochemical parameters or vital organs of the treated animals. No major immune modulatory effects were observed. No signal of genomic content was observed during the supplementation period with the only exception of one cage with two mice which could be due to the continuous "genomic load" during the supplementation period. Taken together, these findings show potential of *P. copri* strain DSM18205 to achieve GRAS status. The results from this safety study in mice are promising and provide the basis for more long-term animal safety studies (chronic toxicity studies), and ultimately clinical trials in humans to investigate *P. copri* as a therapeutic tool in the prevention and cure of the metabolic syndrome.

## Materials and methods

To assess the probiotic effect and safety of *P. copri* administration, mice were gavaged with live or pasteurized *P. copri* for 29 consecutive days. The experimental design of the study is depicted in Fig. 2.

### Culture and treatment of *P. copri*

#### Preparation of *P. copri* for oral gavage

50 ml Peptone Yeast Glucose (PYG) medium was placed in 100 ml serum bottles, sealed with a butyl-rubber stopper, the headspace (the gas phase) gassed with N_2_ gas and bottles autoclaved to achieve sterile anaerobic conditions. The bacteria (*P.copri* strain DSM18205) were grown at 37 °C without agitation. The bacteria were spun down in the logarithmic phase, washed by resuspending in 0.9% NaCl and centrifuged at 3000 rpm for 30 min. The bacterial cell pellet was resuspended in PBS with 10% (vol/vol) glycerol and split equally into 2 tubes for different treatments. Half of the *P. copri* cells were directly aliquoted and frozen at -80 °C for storage, while the other half were pasteurized at 65 °C for 30 min before storing at -80 °C. A representative glycerol stock was thawed and diluted with PBS to determine CFU/ml by plate counting using Schaedler agar plates.

#### Bacterial identification

To assess bacterial contamination and to examine morphological features of the bacteria, gram staining (Sigma-Aldrich) was performed according to the manufacturer’s protocol. Bacteria were identified by quantitative PCR using *P. copri* 16S rRNA gene and universal 16S rRNA gene primers (Table 4)^38,22^.

**Table 4:** Primers used in the qPCR studies.

Gene	Forward primer	Reverse primer	Amplicon length	References
Universal 16S rRNA	ACTCCTACGGGAGGCAGCAGT	ATTACCGCGGCTGCTGGC	192 bp	Scher et al.^22^
*Prevotella copri* 16S rRNA	CGAAAGCTTGCTTTTGATGG	CGCAAGGTTATCCCCAAGT	86 bp	Verbrugghe et al.^38^

### Mouse procedures

#### Mice

30 male 12 week old C57BL/6J mice (Janvier) were housed in controlled conditions (12 h day/night cycle at 22 °C) in a specific pathogen free animal facility. Before the study started the mice were acclimatized for 1 week and split into 15 cages containing 2 mice each. The mice had free access to tap water and standard chow diet. This study was approved by the local Malmö-Lund Ethical Review Committee for Animal Experimentation and conducted in accordance with the European Community regulation concerning the protection of experimental animals (2010/63/EU) and in compliance with the ARRIVE guidelines.

#### Oral gavage

The mice were split into three groups of 10 mice (control, pasteurized and live). The control group received 0.1 ml of a 10% glycerol in PBS solution while the other groups received a dose of 0.1 ml containing 5 × 10^6^ CFU/mL live or pasteurized *P. copri* in 10% glycerol in PBS via intragastric gavaging in the morning for 29 consecutive days.

#### Weighing

Mice were weighed at five time points during the experiment: at baseline, after week 1, 2 and 3 and finally after week 4 on the day of sacrifice (Fig. 2).

#### Faeces, blood and organ collection

Faeces was collected and pooled per cage on day 1, 8, 15, 22 and 29. At sacrifice, the mice were anesthetized with isoflurane and blood collection was performed immediately. Approximately 0.8 ml blood per mouse was collected by cardiac puncture. One part was collected into an EDTA coated tube (Becton Dickinson) (for heamatological analysis), another part was collected into another EDTA coated tube, spun down with supernatant taken off and frozen (for cytokine analysis and LBP ELISA) while a third part was collected in a serum tube (Becton Dickinson), spun down, supernatant taken off and frozen (for biochemical marker analysis). After sacrificing the mice, organs (liver, pancreas, epididymal fat, heart, lung, brain, spleen, kidney and testis) were dissected and relative organ weight was calculated as shown in Eq. 1.

1 $$\mathrm{Relative\, weight\, of\, organ}\left(\mathrm{\%}\right)=\frac{\mathrm{Weight\, of\, organ}}{\mathrm{Live \,body \,weight}}\times 100$$

Equation 1: Calculation of relative weights of organs to bodyweight.

### Oral glucose tolerance test (OGTT)

An oral glucose tolerance test was performed after 7 days of gavaging live or pasteurized bacteria. The mice were fasted for 6 h and 3 g glucose/kg bodyweight was orally administered. Blood glucose content was measured from the tail vein blood before and 15, 30, 45, 60, 90, 120 and 150 min after glucose administration using a HemoCue-glucose analyzer.

### Haematological analysis

EDTA anti-coagulated blood samples were analysed within 7 h with an Abacus Vet5 Hematology analyzer AV5 according to the manufacturer’s instructions.

### Histology

Liver, pancreas, colon, heart, lung, spleen, liver, testis, brain and epididymal fat were dissected, fixed in formaldehyde for 24 h and then stored in 70% ethanol at 4 °C. The organs were embedded into paraffin wax, sectioned 5 µm thick with an Accu-Cut SRM 200 Rotary Microtome, and stained with haematoxylin and eosin. Slides were examined under an Olympus BX60 light microscope.

### Biochemical marker analysis

Serum samples were analyzed using the Cobas 8000 automated platform (Roche) according to the manufacturer's instructions. The following markers were tested: aspartate amino transferase (AST), alanine amino transferase (ALT), alkaline phosphatase (ALP), albumin (ALB), cholesterol, bilirubin, C-reactive protein (CRP), creatinine and blood urea nitrogen (BUN). Samples which showed signs of haemolysis were not included in the analysis.

### Lipopolysaccharide Binding Protein (LBP) ELISA

Mouse plasma was diluted 1:500 and LPS Binding Protein levels were measured with a mouse LBP ELISA kit (Hycult Biotech, The Netherlands) according to the manufacturer’s instructions.

### Cytokine analysis

The cytokines IL-17A (belonging to the mouse Th17 panel 1), IFN-γ, IL-1β, IL-4, IL-6, IL-10 and TNF-α (belonging to the mouse proinflammatory panel 1) (Custom Mouse Cytokine V-Plex kit; cat. No. K152A0H-1) were measured using the Mesoscale Diagnostics (MSD) multi-spot assay system as follows. The standards were reconstituted in the assay diluent provided. The wells were washed three times using 200 μl PBS + 0.05% Tween20, each time soaked for 30 s and then emptied using an Asys Atlantis microplate washer. Standards and samples (diluted ¼ for proinflammatory panel plate, ½ for Th17 panel 1 plate) were added at 50 μl per well, the plates were sealed and incubated for 2 h at room temperature on an orbital shaker (700 rpm, Thermo Scientific). 25 μl per well of the detection antibody was added, the plate sealed and incubated for 1 h at room temperature on an orbital shaker. The plates were washed three times as before. 150 μl of the 2 × MSD Read Buffer T was added to each well and the MSD plates were measured by the Meso QuickPlex SQ 120. The raw data were measured as electro-chemiluminescence signal (light) detected by photodetectors and analysed using the Discovery Workbench 3.0 software (MSD). A 4-parameter logistic fit curve was generated for each analyte using the standards and the concentration of each unknown sample was calculated.

### Quantitative PCR (qPCR)

Total bacterial DNA from faecal samples and caecal content was extracted using the QIAamp PowerFecal DNA Kit (Qiagen) according to the manufacturer’s protocol. DNA concentrations and purity were determined using a Nanodrop spectrophotometer (Nanodrop Technologies) and qPCR analysis was performed using universal 16S and *P. copri* 16S rRNA primers (Table 4)^38,22^. 20 ng of DNA was used in each qPCR amplification, ran in duplicate on the same plate. Detection of the PCR product was carried out by the CFX384 Real-Time PCR system (Biorad) using the DNA-binding dye SYBR Green I (SSo Advanced, Biorad) and the following cycling conditions: 98 °C for 3 min, 40 cycles of 95 °C for 15 s, and 62 °C for 15 s.

### Statistical analysis

ANOVA general linear model followed by a Tuckey’s post hoc test was performed using a commercial software package (GraphPad Prism).

## Supplementary Information

Supplementary Information.

## Data Availability

The data that support the findings of this study are available from the corresponding author upon reasonable request.
