# Supplementary material for: Evaluation of hypoglycemic effect, safety and immunomodulation of Prevotella copri in mice
Source: Sci Rep. 2021 Oct 28;11:21279. doi: 10.1038/s41598-021-96161-6 (PMC8553810; doi:10.1038/s41598-021-96161-6)
Supplement: Supplementary file 1 — Supplementary Information. [file 41598_2021_96161_MOESM1_ESM.docx]

**Supplementary information**

**Evaluation of hypoglycemic effect, safety and immunomodulation of *Prevotella copri* in mice**

Phebe Verbrugghe^1,*^, Jón Brynjólfsson^1^, Xingjun Jing^1^, Inger Björck^2^, Frida Hållenius^1^, Anne Nilsson^1,*^

^*^ Correspondence: [vphebe4@yahoo.com](file:///C:\Users\P&O%20Enterprises\Dropbox\Phebe\food%20health\Publications\PUBLICATIONS\Publication%20mouse%20safety%20study\Scientific%20Reports\vphebe4@yahoo.com), [anne.nilsson@food.lth.se](mailto:anne.nilsson@food.lth.se)

^1^Food Technology, Engineering and Nutrition, Lund University, PO Box 124, 221 00 Lund, Sweden

^2^Food for Health Science Centre, Lund University, PO Box 124, 221 00 Lund, Sweden. Present affiliation ProPrev AB, 254 40 Helsingborg, Sweden

**
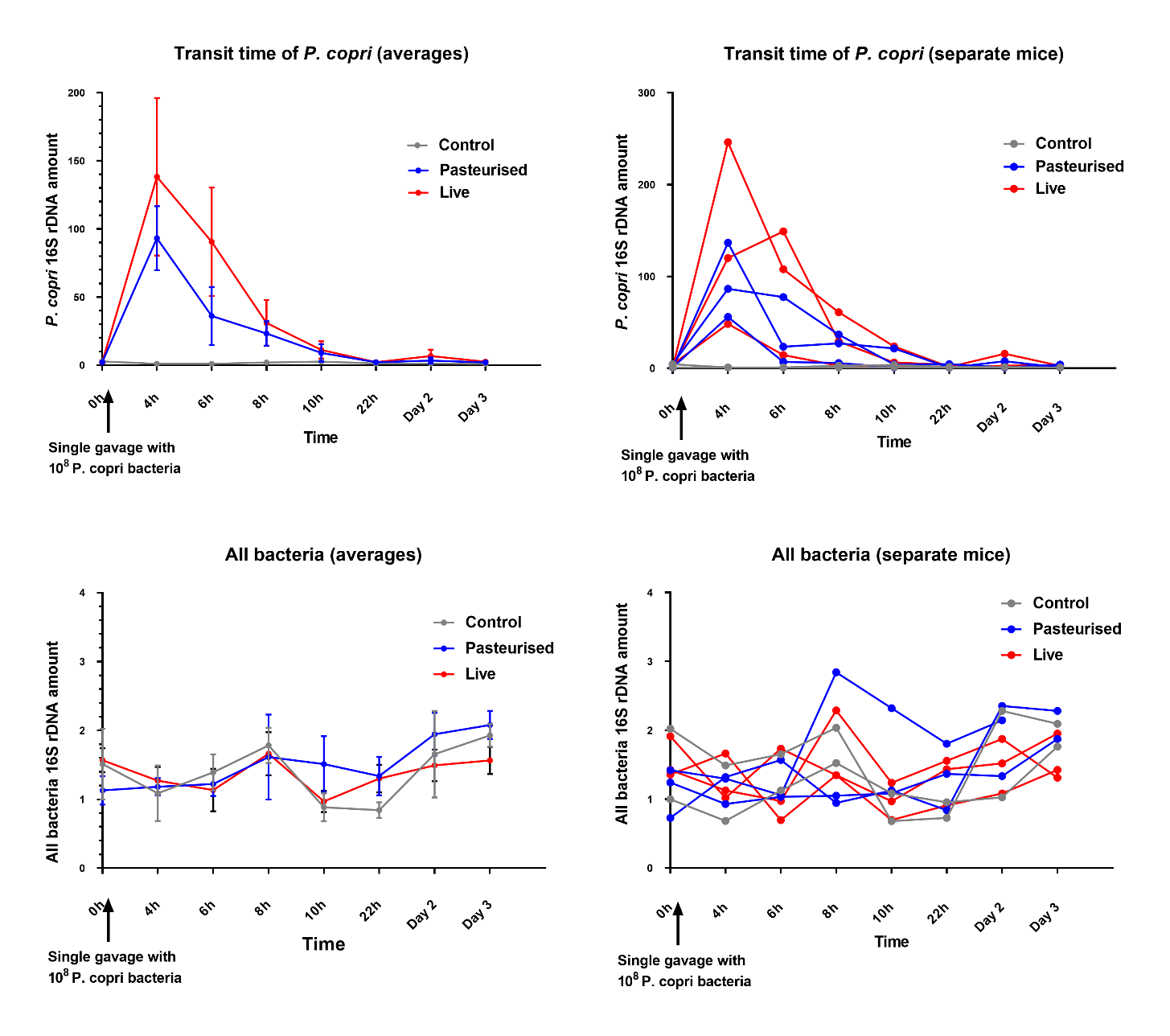
**

**Supplementary figure 1.** Transit time of *P. copri* (top) after one single gavage of 10^8^ *P. copri* bacteria. *P. copri* and all bacteria 16 rDNA amount as determined by qPCR: average (left) and for separate mice (right).
